# MWCNTs dispersion adopting GA and its application towards copper tailings-based cementitious materials

**DOI:** 10.1038/s41598-023-43133-7

**Published:** 2023-09-26

**Authors:** Bingzhi Xiang, Ruifeng Cheng, Jielu Zhu, Yong Zhou, Xiaoying Peng, Junwei Song, Junhong Wu

**Affiliations:** School of Urban Construction, Jiangxi University of Technology, Nanchang, 330098 China

**Keywords:** Other nanotechnology, Composites, Glasses, Mechanical properties, Nanoscale materials, Carbon nanotubes and fullerenes

## Abstract

Hydrophobic carbon nanotubes are hardly to disperse in water and prone to agglomerate when poured with Copper Tailing-Based Cementitious Material (CTCM). Multi-walled carbon nanotubes (MWCNTs) + Arabic Gum (GA) dispersions were prepared by a novel method of synergistic optimization of concentration, controlling low-frequency ultrasonic time and setting the ambient temperature with non-toxic anionic surfactant GA as surfactant. The results of UV–Vis spectroscopy showed that the high stability MWCNTs + GA dispersion with low aggregation area (< 1.2%) and low aggregation beam size (< 219 nm) have been prepared by using 1.7 mmol/l GA. The effects of highly stable MWCNTs dispersion on the mechanical properties, microstructure and durability of CTCM were studied. The 28 days compressive strength increased by 21.5%, and the flexural strength increased by 20.5%, almost reaching the mechanical level of the control group. The results of SEM, XRD and EDS showed that GA significantly enhanced the dispersion of MWCNT in aqueous solution at a suitable concentration (mass ratio of GA:CNTs = 1:1). The microstructure of the prepared CTCM by high stability MWCNTs dispersion was optimized obviously, and the mechanical properties and durability were improved significantly. This method solves the dual problem of MWCNTs not being fully dispersed in aqueous solution and being easily re-agglomerated in cementitious materials, as well as finding a breakthrough for the low cost and industrialization of tailings cement-based composite cementitious materials.

## Introduction

Copper tailings are fine powder left after crushing, grinding and cleaning of copper ore. As an industrial waste, it is seriously accumulated in some countries and has caused serious environmental problems. There are many industrial wastes, such as waste glass^1,2^ and solid waste from mining and metallurgy, which can be recycled as building materials. Grinding to the specified fineness of copper tailings powder with aluminosilicate as the main component can replace part of cement to prepare CTCM, and can also completely replace cement to prepare geopolymer concrete (GPC)^3^. CTCM is an inorganic composite material formed by mixing slag-based cementitious materials, copper tailings powder, and admixtures. CTCM recycles copper tailings as construction engineering materials, helping to reduce carbon dioxide emissions (carbon emission reduction) from the building materials industry^4^.

However, CTCM inevitably produces microcracks during the service life. The expansion of microcracks and even the development of fractures will destroy the integrity of concrete structures and reduce durability. Dispersing carbon nanotubes (CNTs) and other nanomaterials in cement-based materials to develop crack-free and durable building materials has been studied by scholars^5–8^. The theoretical strength and elastic strain capacity of CNTs are 100 times and 60 times that of steel, respectively^9^. These advantages make CNTs ideal nano-reinforced materials for the development of high-strength, high-performance composites^10–15^. Nowadays, researchers have used CNTs as nano-reinforced materials for ceramic and cement composite cementitious materials^16–20^.

CNTs are composed of rolled single or multi-layer graphene sheets. Each carbon atom of the nanotube combines with the surrounding three carbon atoms through sp2 hybridization, forming a hexagonal plane to form a cylindrical surface. MWCNTs are made of more concentric graphite layers around the central tube, while single-walled carbon nanotubes (SWCNT) are composed of a single graphite cylinder^21,22^. Therefore, MWCNTs have many potential applications and excellent properties, with Young's modulus of about 1.0 TPa and tensile strength of 50–200 GPa^23–25^. The unique structure determines the thermodynamic, chemical, optical, electrical and mechanical properties of MWCNTs^26–30^.

MWCNTs are ideal reinforcement materials for manufacturing high-performance composite cementitious materials. The hydration products of CTCM have typical C-A-S-H gel structure. The interface between MWCNTs and C-A-S-H gel structure has excellent mechanical properties, especially the well dispersed MWCNTs, which directly determines the efficiency of copper tailings powder modified cementitious materials. CTCM is a composite cementitious material after replacing part (15–30%) of cement with copper tailings powder. Its mechanical properties are significantly lower than those of the same grade cement. After being enhanced by MWCNTs, its mechanical properties are restored or enhanced compared with the same grade pure cement. The excellent mechanical properties of MWCNTs nano-scale are transferred to CTCM composite cementitious materials.

The suitable active dispersants and dispersion procedures are required to ensure the good and stable dispersion of CNTs in the matrix and to successfully transfer the excellent mechanical properties of CNTs into composite cementitious materials. CNTs have a great propensity to aggregate due to van der Waals force. As a result, it takes the right procedure and tools to prepare a homogeneous CNTs dispersion suspension in cement paste, but it is still a challenging problem to achieve uniform and stable CNTs dispersion in cement paste. The typical method for combining CNTs with cement matrix is to first disperse these CNTs in water^31–33^, and then combine carbon nanomaterials and water suspension with cement powder using a standard mixer. This method helps to avoid agglomeration^34–36^. To make homogenous aqueous dispersions of CNTs, a variety of physical and chemical methods are employed^37–39^, including ultrasonic treatment, mechanical stirring, the use of surfactants and polymers^40,41^, CNTs functionalization, etc. The non-covalent functional dispersion approach using surfactants, in particular, provides better application value without altering the natural electrical, optical, or mechanical properties of CNTs. PVP, Triton X-100, SDBS, Pluronic-F127, and GA, etc. are some of the surfactants commonly used in this approach to disperse CNTs. While GA, cheap, non-toxic, simple dispersion process, stable dispersion, in these surfactants by means of ultrasonic treatment to decompose or unbind CNTs polymers, the best results. This surfactant has obvious effects on the decomposition or dissociation of CNTs by ultrasonic treatment. Among these surfactants, GA is a safe and harmless food thickening additive with good biocompatibility and is particularly favored^42^. Very low concentrations of CNTs aqueous dispersions have been studied using GA for a variety of applications^43–47^.

The objective of this study is to gain insight into how CNTs are better dispersed in CTCMs and can fully exert their nanoscale enhancement, expecting to achieve the level of Ultra-high-performance concrete (UHPC)^48^. In this study, the technology of GA-assisted dispersion of CNTs and the effect of its dispersion liquid on the overall mechanical properties of the slurry were first studied experimentally, and the effect of CNTs on local microcracks of cement slurry was studied by SEM, XRD, and EDS. The results show that CNTs can change the fracture failure mode and enhance the cement matrix at the local crack by bridging fracture mechanism, thereby improving the overall mechanical properties. In addition, the mechanism of GA-assisted dispersion of CNTs was discussed and analyzed by UV–visible spectroscopy and optical microscope image analysis. The presented data can be applied to future CTCM research. CTCM is an innovative nanocomposite material with high environmental and economic benefits, which can greatly reduce environmental pollution and waste of resources. CTCM is a mining and metallurgy solid waste-based cementitious material reinforced by carbon nanomaterials, which expands a new field of reinforced solid waste-based concrete.

### Materials

**Figure 1:**
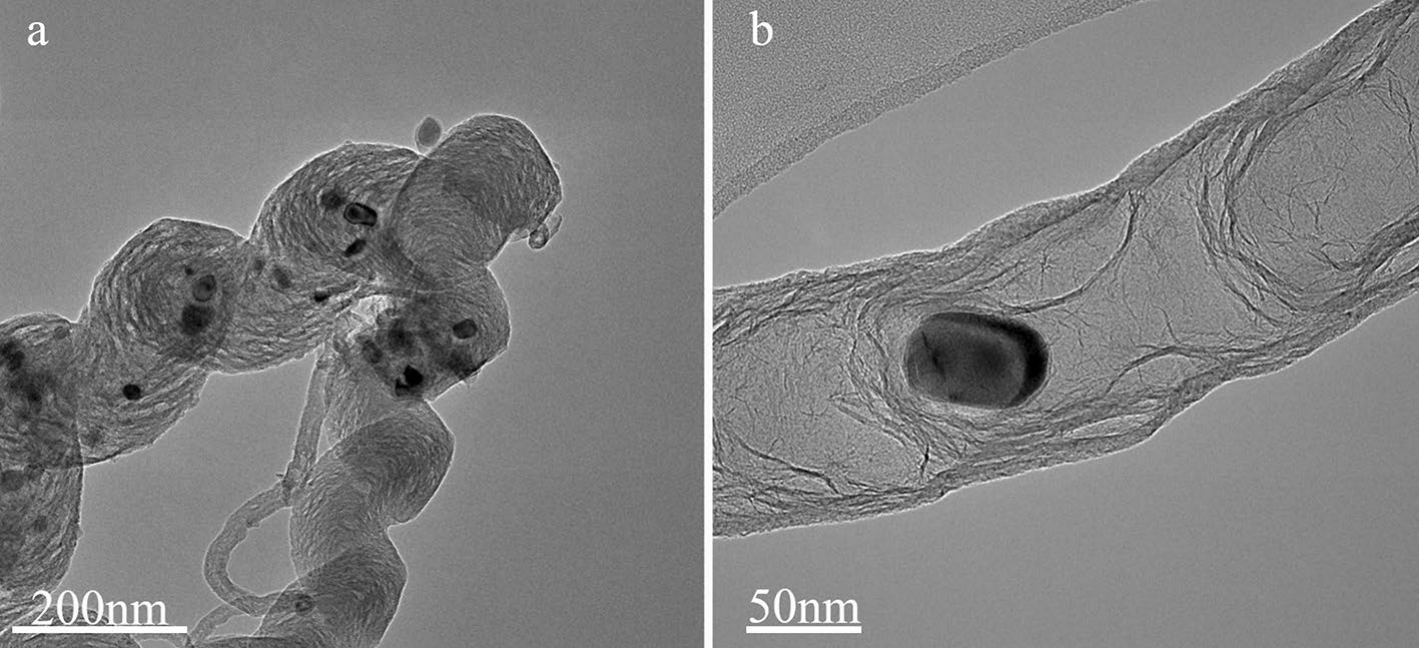
TEM images of MWCNTs. (**a**) 200 nm and (**b**) 50 nm (Provided by Nanotech Port Corporation).

MWCNTs were purchased from Nanotech Port Corporation (NTP, Shenzhen, China). The physical properties are listed in Table 1. Figure 1 is the TEM (Tecnai G2 Spirit, FEI Co.) image of MWCNTs, from which the morphology of MWCNTs can be roughly estimated. The anionic surfactant used in this study was GA (C_12_H_7_ClN_2_O_3_, AR, molecular weight 2.5 × 10^5^–1 × 10^6^), purchased from Sinopharm Chemical Reagent Co., Ltd. (Shanghai, China). The components of GA are as follows: d-galactose 44%, l-arabinose 24%, d-glucuronic acid 14.5%, l-rhamnose 13%, 4-*O*-methyl-d-Glucuronic acid 1.5%, protein 2%, impurities 1%. The dispersion medium is distilled water.

**Table 1:** The physical parameters of MWCNTs.

Products	Outer diameter/nm	Length/μm	Purity	Specific surface area/m^2^ g^−1^
L-MWCNT	20–40	5–15	99	90–120

**Figure 2:**
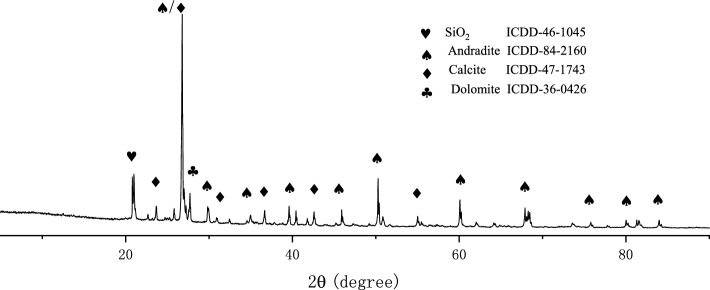
XRD pattern of copper tailings powder.

The experimental cement is P.O. 42.5 (OPC), which was provided by Jiangxi WanNianQing Cement Co., Ltd. The copper tailings powder (CTP) was provided by Jiangxi Copper Group and dried at 80 °C for 24 h. The chemical composition of the two materials was tested by X-ray fluorescence analysis, and the chemical composition is shown in Table 2. It can be seen from the table that the copper tailings powder contains a large amount of CaO, SiO_2_ and Al_2_O_3_. According to ASTM C618-15 Standard Specification for Natural Volcanic Ash Raw Materials or Calcined Materials for Cement and Concrete, a necessary condition for volcanic ash materials is that the total amount of SiO_2_, Al_2_O_3_ and Fe_2_O_3_ should exceed 70% while the total amount of copper tailings powder is 42.08%, far from meeting the standard requirements. The XRD (Rigaku Smart Lab SE, Japan, 10–90°, 2°/min, Cu-Kα ray, 40 kV, 40 mA) test results (Fig. 2) show that SiO_2_ mainly exists in the form of andradite, rather than in the form of active SiO_2_. The poor activity of this copper tailings can be seen from the components in this study.

**Table 2:** Chemical composition of cement and copper tailings powder (wt, %).

Composition	CaO	MgO	Al_2_O_3_	SiO_2_	Fe_2_O_3_	SO_3_	P_2_O_5_	Na_2_O	K_2_O	CuO	MnO	LOSS
OPC	60.71	0.88	5.20	21.18	4.44	3.59	0.10	0.12	0.71	0.04	0.06	2.97
CTP	31.76	5.37	5.49	28.2	8.39	7.2	0.11	0.67	1.31	0.08	0.13	7.29

### Experiments

#### MWCNTs and GA dispersion preparation

MWCNTs nanofluids were prepared by using six concentrations of GA active agent, and they were compared to a standard solution (absorbance 1) and a sample of pure water (absorbance 0). The mass ratio of surfactant GA to MWCNT was used to prepare the surfactant solution (1:1, 2:1, 3:1, 4:1, 5:1, 6:1). The surfactant concentration of GA ranged from 1.7 to 10.2 mmol/L. The material composition of cement blank sample and CTCM sample is shown in Table 3.

**Table 3:** Ratio setting of composite cementitious materials (mass fraction/%).

Samples	Cement	CTP	GA	MWCNTs	W/B
C0	1	0	0	0	0.4
C-CT	0.75	0.25	0	0	0.4
G1	0.75	0.25	0.08	0.08	0.4
G2	0.75	0.25	0.16	0.08	0.4
G3	0.75	0.25	0.24	0.08	0.4
G4	0.75	0.25	0.32	0.08	0.4
G5	0.75	0.25	0.40	0.08	0.4
G6	0.75	0.25	0.48	0.08	0.4

**Figure 3:**
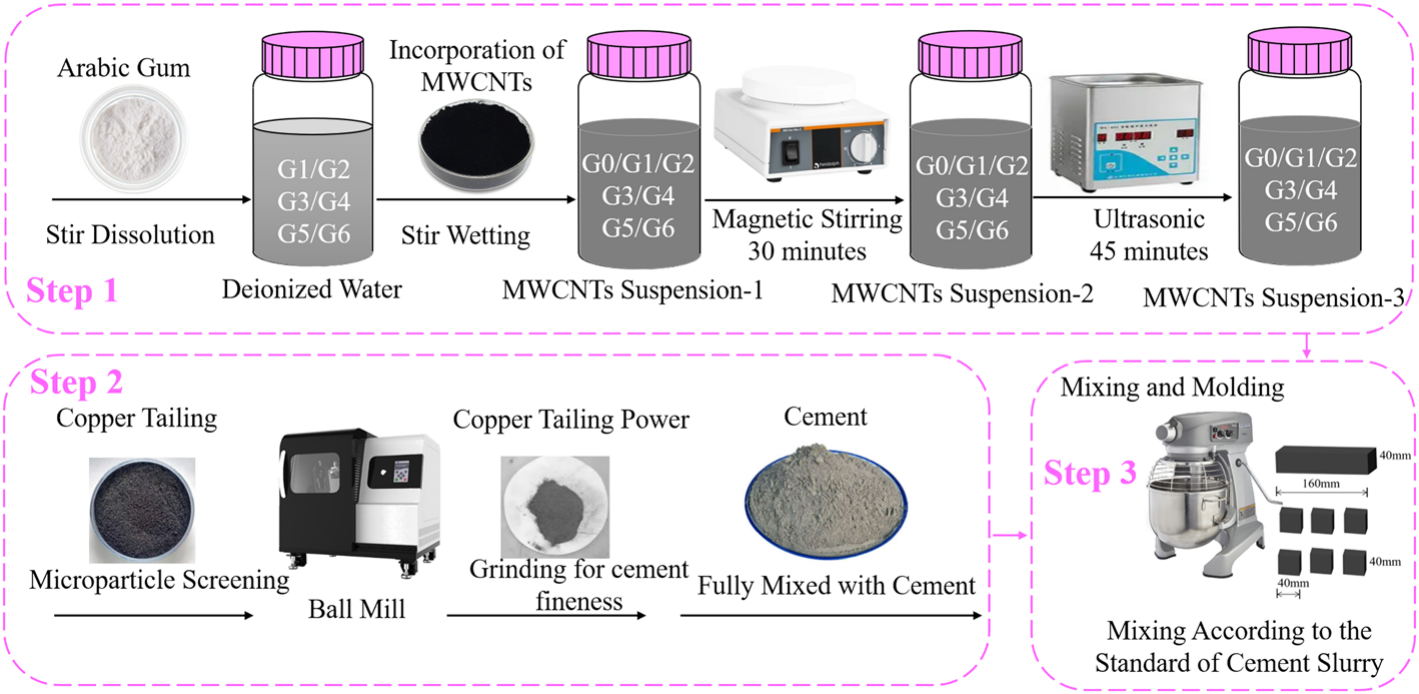
Preparation process of MWCNTs dispersion and specimens.

Six different concentrations of GA surfactant solutions were prepared while maintaining the MWCNT content at 0.16 g/L. The samples were dispersed ultrasonically in an ultrasonic cleaner with a frequency of 120 kHz and a power of 600 W, all the while keeping the temperature constant at 25 °C. After 45 min of ultrasonic dispersion, all samples were spun using a H/T16MM high-speed centrifuge for 30 min at 2000 RPM and room temperature (25 °C) to separate the MWCNT attachments. The schematic diagram is displayed in Fig. 3. After centrifugation, the supernatant was separated from the precipitate, and the supernatant was used for further analysis.

**Figure 4:**
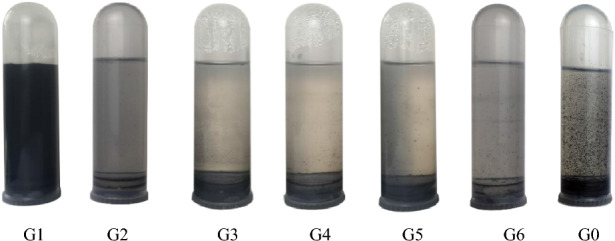
MWCNTs dispersions standing for 60 days.

Six MWCNT dispersion samples with different concentrations of GA and MWCNT dispersion sample without surfactant were standing for 60 days, as shown in Fig. 4.

The sample numbers G1, G2, G3, G4, G5 and G6 represent the mass ratio of GA: MWCNTs = 1:1, 2:1, 3:1, 4:1, 5:1, 6:1, respectively. G0 represents the sample without active dispersant.

#### Preparation of MWCNT reinforced copper tailings powder cement sample

The prepared MWCNTs and GA dispersion were poured into a cement paste mixing pot, and the mixed copper tailings powder cement (copper tailings powder/cement mass ratio 1:3) was added to the mixing pot. Note that when preparing MWCNTs reinforced cement pastes, ultrasonic power (600 W) is set to achieve sufficient ultrasonic energy for the dispersion of MWCNTs. After ultrasonic treatment, cement slurry mixer was used according to GB / T17671-2021(Chinese standard).

Cement and copper tailings powder were combined to create a blended powder, which was then combined with the prepared MWCNTs dispersion. The following is the mixing procedure: The dispersion should first be added to the mixing bowl and pre-stirred for 60 s. After that, add to the pot containing the copper tailings powder cement and stir gradually for 180 s, or until the cement slurry seems uniform. The MWCNTs were then successfully further dispersed by being agitated at high speed for 120 s in the cement slurry. The water cement ratio of all six batches of samples was 0.4.

Each batch of MWCNTs-copper tailings powder cement paste samples with different mixing designs were uniformly stirred and cast into cubes with a size of 40 × 40 × 40 mm^3^ and rectangles with a size of 40 × 40 × 160 mm^3^. All samples were removed from the mold after 24 h and transferred to the standard curing room (20 ± 2 °C, RH > 95%) for curing. According to the Chinese standard GB/T17671-2021, the compressive test was carried out with a cube sample of 40 × 40 × 40 mm^3^ and the bending test was carried out with a sample of 40 × 40 × 160 mm^3^. The compression and bending strength were tested at 3, 7 and 28 days, respectively. The loading rates were set to 2.4 kN/s and 50 N/s, respectively. The test instrument is HYZ-300 constant loading cement flexural and compressive test machine.

In addition, the microstructure and composition of the corresponding age were tested in turn. Microstructure study using a scanning electron microscope (SEM, FEI Quanta 650 FEG); x-ray powder diffraction (XRD) data were measured on a Bruker D8 Advance diffractometer.

**Figure 5:**
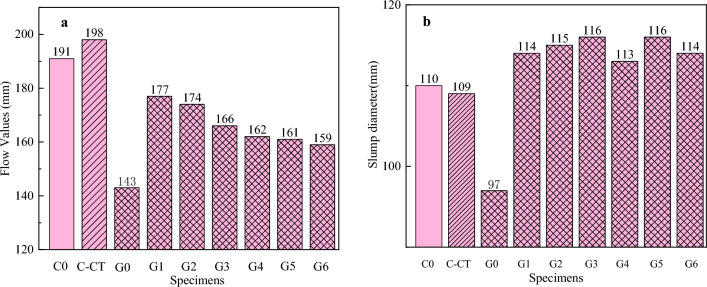
The working performance. (**a**) Fluidity of cementitious mixture paste. (**b**) Slump values of cementitious composites.

According to the GB/T 8077-2012 (Chinese standard), the fluidity of cement paste is measured, and the results are as follows Fig. 5a. After the fluidity test, the above mixed cement gel was prepared again, and the corresponding concrete was configured according to the laboratory mix ratio, poured into the mold and vibrated to compact, and the slump was tested. According to the 'concrete quality control standard' GB 50164-2011 (Chinese standard), the obtained concrete samples were evenly loaded into the slump cylinder in three layers with a small shovel, so that the height of each layer was 1/3 of the height of the cylinder after tamping. Each layer was mashed 25 times with a ramming rod. The lift-off process of the slump cone should be completed within 3–7 s; the whole process from the beginning of loading to the lifting of the slump cylinder should be carried out uninterruptedly and should be completed within 150 s. The test results are shown in Fig. 5b.

### Characterization

#### Ultraviolet–visible spectrum of MWCNTs suspension

The MWCNTs distributed in the aqueous suspension are active in the UV–Vis region during the UV–Vis spectrum test because of the extra absorption brought on by the 1D van Hove singularity^49–53^.

**Figure 6:**
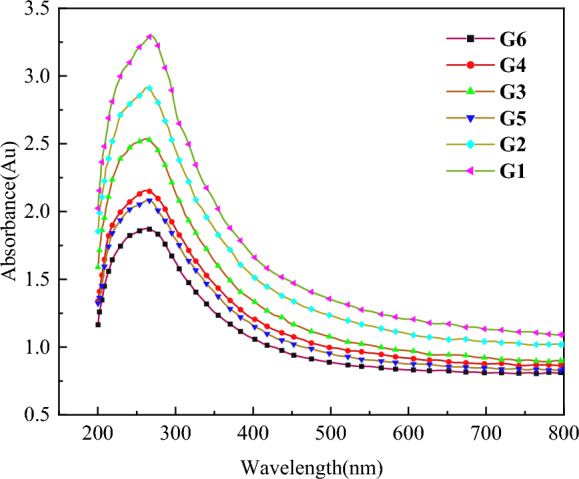
Ultraviolet visible absorption spectrum curve of CNTs suspension prepared by GA.

Figure 6 shows the UV–Vis absorption spectra of MWCNT suspensions made with various GA surfactant concentrations (0.16–0.64 g/L), 45 min of ultrasonic duration, and constant temperature (25 °C). The UV–visible absorbance correlates to the p-electron transition of the carbon band in CNTs and represents the usual characteristic peaks of all spectra near 260 nm. The sharpness of the absorbance spectral band rises when CNTs are evenly spread^54,55^.

G1–G6 aqueous solution has an absorbance of approximately 3.6, 2.9, 2.5, 2.2, 2.1, and 1.9 Au, respectively. The outcomes demonstrated that, under specific ultrasonic treatment time and temperature conditions, the dispersion of MWCNTs in suspension decreased as the concentration of GA surfactant increased, indicating that a high concentration of GA surfactant was ineffective in enhancing MWCNT dispersion.

The agglomerated carbon nanotube bundles were peeled off into different tubes or thinner bundles for a period of time by water bath and ultrasonic treatment, and then stabilized by the space filling force and electrostatic repulsion of the surfactant.

The Lambert–Beer's law is described by the equation:

1 $$A = \log \left( \frac{1}{T} \right) = \log \left( {\frac{{I_{r} }}{{I_{S} }}} \right) = Ecl$$

where, A is the absorbance of MWCNTs suspension, T is the transmittance, E is the absorption coefficient, c is the concentration of MWCNTs suspension, and l represents the optical path length.

The absorbance A of MWCNTs suspension is directly proportional to the product of concentration c and optical path 1 of the solution. Then the concentration of MWCNTs suspension can be calculated by measuring the absorbance value. Therefore, the effect of different concentrations of dispersant on MWCNTs suspension can be determined.

The preparation concentration of MWCNTs suspension is 0.08 g/L, the concentration of dispersant is 0.45 g/L, and the ultrasonic treatment lasts for 30 min. Use an ultraviolet/visible spectrophotometer to measure the absorbance at a wavelength of 260 nm. Prepare an aqueous solution of dispersant with the same concentration as the reference solution. To measure the transmittance of MWCNTs solution at a constant room temperature, dilute the supernatant of six sample solutions by 100 times and take deionized water as the reference solution. Then, pour the MWCNTs solution before and after absorption into the quartz cuvette and use a UV-560 ultraviolet/visible spectrophotometer to measure the transmittance.

It is described by the following equation:

2 $${\text{A}} = {3}.{\text{46848B}} + 0.{17497}$$

wherein, B represents the concentration of functionalized MWCNTs solution, A represents the absorbance value. According to the fitting equation, measure the ultraviolet absorption rate of MWCNTs suspension, and check the concentration of the dispersed MWCNTs. The influence of different concentration of GA dispersant on the dispersion of MWCNTs in aqueous solution can be characterized by the concentration change of MWCNTs. As shown in the Fig. 6, It was found that the absorbance of G1 group was higher than that of other control groups. p–p stacking interaction, there is a strong bonding interaction between the benzene ring of GA and MWCNTs, and hydroxyl groups attract water molecules. The electrostatic repulsive force and steric hindrance of GA together reduce the surface energy of MWCNTs, thus improving the dispersion of the solution.

#### Effect of MWCNT on the fluidity of cement slurry

Working performance is a key index to evaluate the storage and transportation of fresh cement paste. According to this index, the fluidity should be distinguished according to the concentration of GA-MWCNT suspension, that is, when the higher concentration of GA-MWCNT suspension is used, the fluidity of the paste is relatively small. On the contrary, when using a lower concentration of GA-MWCNT suspension, the slurry fluidity is relatively large. According to the slump specified in the concrete pumping agent JCJ/T 10-2011 'Concrete Pumping Agent' standard, we used the slump test to study the effect of MWCNT on the fluidity of cement slurry. The results are shown in Fig. 5b.

**Figure 7:**
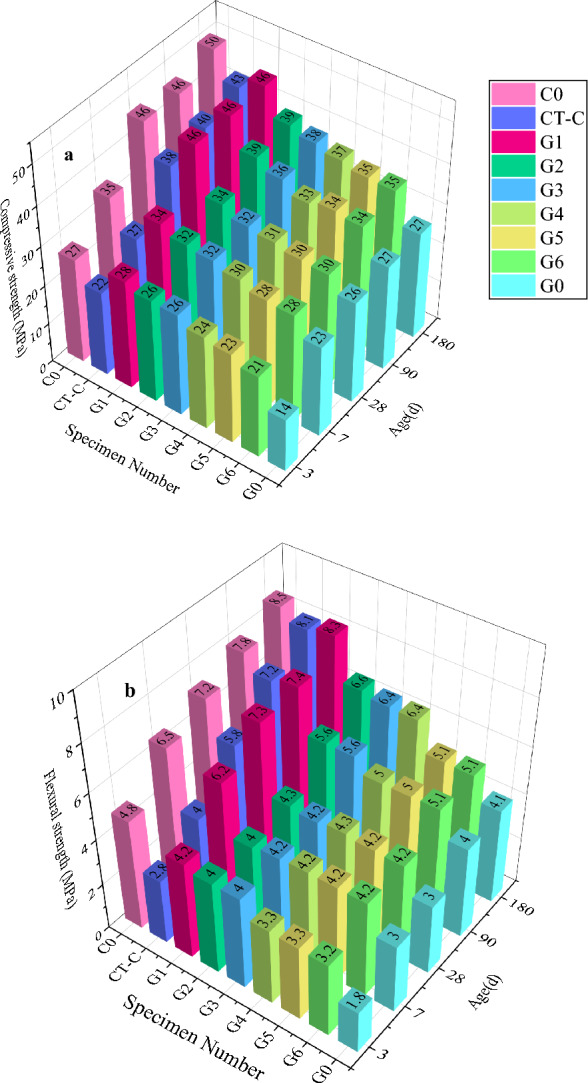
Mechanical properties of the samples. (**a**) Compressive strength of MWCNTs incorporating cementitious composites after 3, 7, 28, 90 and 180 days of the hydration period. (**b**) Flexural strength of MWCNTs incorporating cementitious composites after 3, 7, 28, 90 and 180 days of the hydration period.

Most previous studies have shown that due to the high specific surface energy of MWCNT, the addition of MWCNTs reduces the machinability of cement-based composites. In this study, the influence of undispersed MWCNTs on the fluidity of cement slurry was also tested. Six MWCNTs with different concentrations of active dispersants (surfactant/CNT mass ratio 1:1, 2:1, 3:1, 4:1, 5:1, 6:1) were considered, and compared with blank samples without active agents, the water binder ratio was controlled to 0.4.

There is no standard value reference for the fluidity of cement paste, which is affected by many factors. Only the approximate range can be tested to ensure that it can meet the requirements of related projects. The flow values of pure cement paste (C0), copper tailing powder cement paste (C-CT), MWCNTs without dispersant, copper tailing powder cement paste (G0) and MWCNTs with different concentrations of GA dispersant, copper tailing powder cement paste (GA1 ~ GA6) cement-based composites are shown in Fig. 5a. From the data in the figure, it can be seen that the presence of MWCNTs will reduce the fluidity of cement-based composite paste.

**Figure 8:**
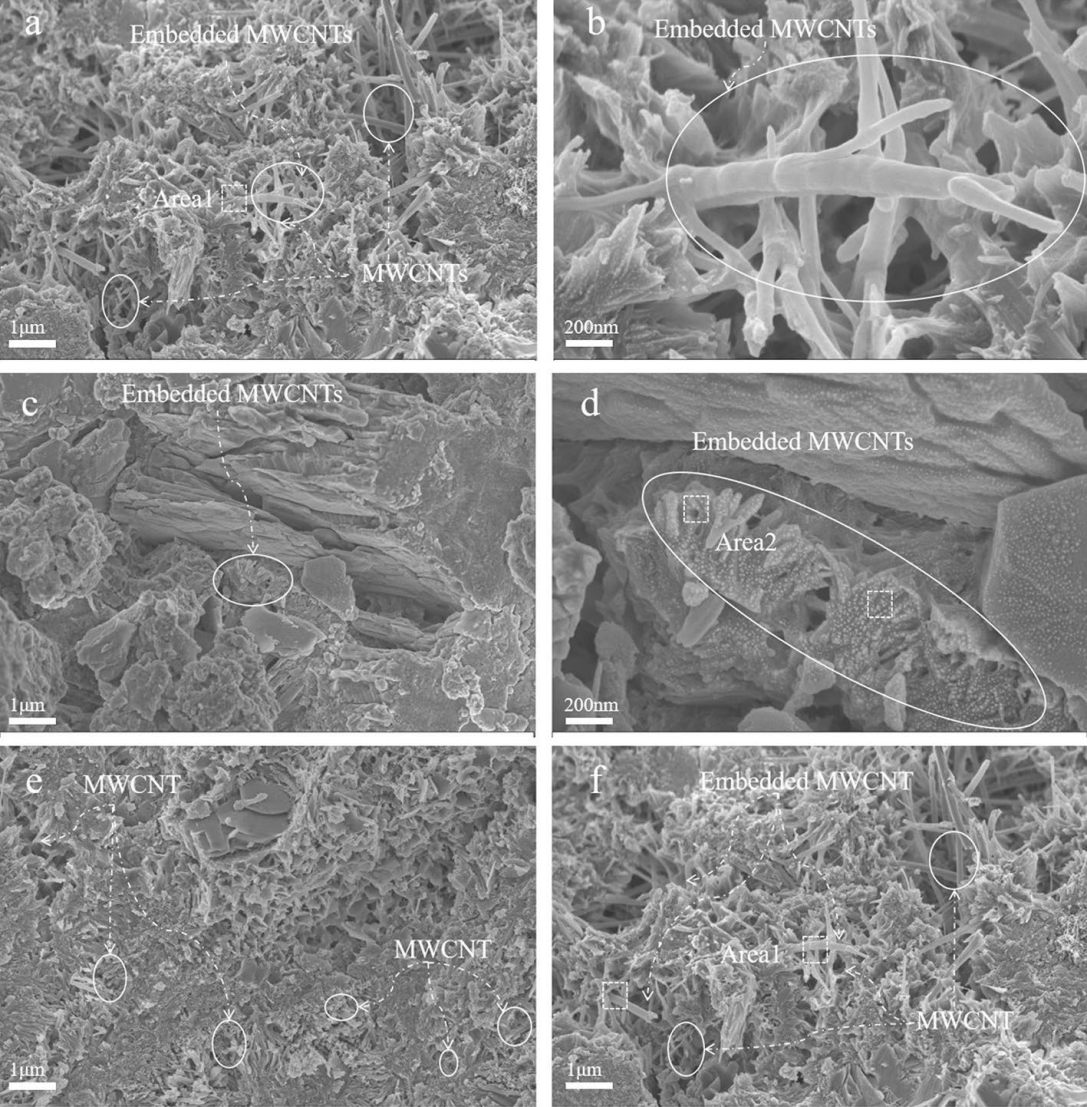
SEM morphology of the MWCNTs-CTCM sample after the mechanical strength measurement (the areas with white square markings in the SEM images are selected areas for EDS detection).

The fluidity of MWCNTs copper tailings powder cement paste G1, G2, G3, G4, G5 and G6 is reduced by 7%, 9%, 13%, 15%, 15.7% and 16.7% respectively compared with pure cement paste C0, and the fluidity is reduced by 10.6%, 12%, 16%, 18%, 18.7% and 19.7% respectively compared with the C-CT without MWCNT, while compared with G0, the fluidity was increased by 23.8%, 21.6%, 16.1%, 13.3%, 12.6% and 11.2% respectively. The use of GA active dispersant increased the flow value of cementitious materials significantly. This is consistent with literature^35^. MWCNTs can adsorb water molecules due to their large specific surface area. The content of free water required for the fluidity of cement paste is significantly reduced, resulting in reduced fluidity.

#### Mechanical properties

On the mechanical properties of cement pastes, the impacts of MWCNTs dispersions with various concentrations of GA were examined. In Fig. 7, the test results are displayed. The significant improvement in strength of CTCM suggests that the active dispersant GA is successful in dispersing MWCNTs in CTCM matrix and that the mass ratio with MWCNTs is appropriate.

The compressive strength of each specimen is shown in Fig. 7a at 3, 7, 28, 90, and 180 days. The C-CT group was examined first. Due to the addition of 25% copper tailings powder with insufficient activity, the compressive strength of 3, 7, 28, 90, and 180 days dropped by 19.1%, 21.1%, 17.4%, 12.2%, and 13.3%, respectively, when compared to the blank group C0.

At 3, 7, 28, 90, and 180 days, the compressive strength of the G1 group was higher than that of the CT-C group by 29.1%, 25.6%, 21.5%, 14.3%, and 7.3%, respectively.

And then, G1 compared to the control group C0, the maximum difference in short-term compressive strength was 4.3%, while the maximum difference in long-term compressive strength was 7%. However, compared to the C-CT group, the compressive strength of the G0 group at 3, 7, 28, 90, and 180 days was decreased by 36.6%, 14.7%, 31.7%, 33.2%, and 37.0%, respectively. This is because the G0 group had a significant amount of carbon nanotubes but no GA dispersing agent.

The flexural strength of CT-C group is shown in Fig. 7b, and the flexural strength of 3, 7, 28, 90, and 180 days dropped by 41.7%, 38.5%, 19.4%, 7.7%, and 2.4%, respectively, when compared to the blank group C0. However, the flexural strength of the G1 group at 3, 7, 28, 90, and 180 days, was higher than that of the CT-C group by 33.3%, 35.5%, 20.5%, 2.7%, and 2.4%, respectively.

From Fig. 7b, it is clear that the flexural strength of the G1 group increased significantly with the addition of MWCNTs. The strength difference of these sample groups shows that the effectively dispersed MWCNT will play a strengthening role, making up for the strength loss of CTCM due to the replacement of 25% cement by copper tailings powder.

And both the short-term and long-term compressive strength decreased significantly, indicating that the addition of carbon nanotubes, if poorly diffused or non-diffused, has little effect on the strength and has a negative effect. Combined with the strength change data of G2 to G6 sample groups, it can be seen that GA active dispersant plays a decisive role.

The amount of surfactant will directly affect the performance of MWCNTs. However, a large amount of active agent components will also reduce the compressive strength of cement paste. Because the use of GA leads to foam formation and makes the matrix more porous, the use of defoamer (TBP) can alleviate the influence of this factor^47^. Carbon nanotubes dispersed in surfactant (GA) also have critical concentration or critical concentration region^49^. After exceeding the threshold concentration, increasing the GA concentration may lead to more MWCNTs aggregation, which will weaken the interfacial interaction between MWCNTs and cement hydration^50^.

The macroscopic defects inevitably caused in the mixing process have the greatest influence on the flexural strength of cement paste. MWCNTs is a perfect two-dimensional nanofiber material, which can improve the fracture resistance of cement matrix by offsetting microcracks and avoiding their fusion into larger microcracks.

#### Microstructure and morphology

The mechanical properties of cement paste after curing are affected by its microstructure characteristics. After 28 days, the final hydration products of cement were almost indistinguishable from MWCNTs. However, at 7 days, the dispersion and state of MWCNTs in the cement slurry can be observed.

**Figure 9:**
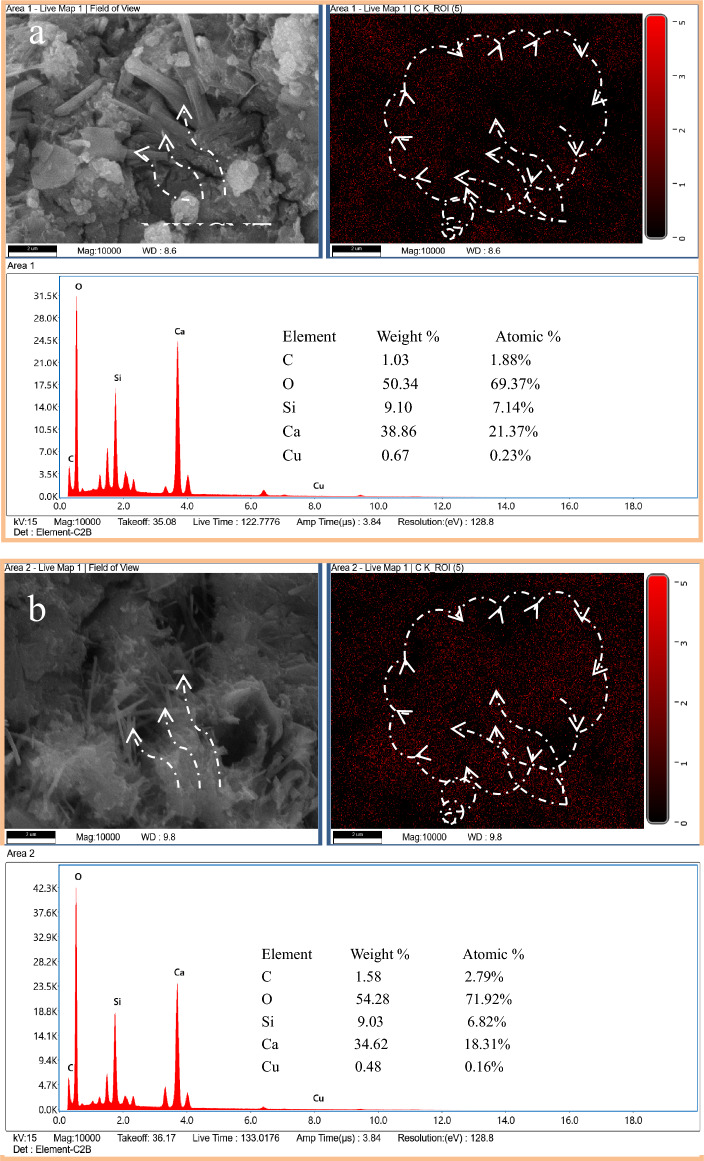
The result of EDS detection: (**a**) Area1 EDS detection and (**b**) Area 2 EDS detection.

The microstructure of needle-like ettringite is similar to that of MWCNTs, and the existence of MWCNTs is proved by EDS analysis. The area selected for EDS detection is represented by the box, and the results are shown in Fig. 9. Large amounts of calcium, silicon and oxygen may be detected in the Area1 and Area2 regions. This is because the GA active functional groups on the surface of MWCNTs promote the synthesis of hydration products. Figure 8 shows the SEM images of MWCNTs in CTCM matrix. MWCNTs with a length of less than 3 μm are uniformly dispersed throughout the hydrate. Because MWCNTs were well dispersed in GA1 samples, no evidence of MWCNTs aggregation was found. The fracture surface of the whole matrix is covered with many MWCNTs, and the fracture is visible and attached to the matrix.

The bridging effect of carbon nanotubes is represented by a small number of unbroken and connected MWCNTs between cracks. Some dispersed carbon nanotubes are pulled out at one end and anchored in the matrix at the other end. These morphologies indicate that the concentration of the active dispersant of the G1 sample and the mass ratio to MWCNTs are appropriate and the dispersion is effective. Therefore, the better the dispersion of MWCNTs in CTCM, the less affected by stirring and pouring.

The fracture of MWCNTs in the CTCM matrix indicates that there is a high bonding strength between MWCNTs and the surrounding matrix, as shown in Fig. 8. However, MWCNTs are also pulled out due to load transfer when the strength of certain sections of the CTCM matrix is less than the yield strength of MWCNTs, as shown in Fig. 8a.

At 7 days of age, MWCNTs were mostly found dispersed into a single root in the G1 and G2 groups (Fig. 8b,c), indicating that the dispersion was effective and stable. Furthermore, MWCNTs play a nucleation role in the cement hydration process and expand along the radial direction of MWCNTs, promoting the growth of hydration products. The MWCNTs (GA/CNTs mass ratio 1:1) dispersed by GA solution after ultrasonic treatment obtain satisfactory dispersion, as shown in Fig. 8c.

Even with changes in the composition of the Copper tailings cementitious material (CTCM) matrix, the crack bridging effect of MWCNTs is still evident in the SEM analysis, underscoring the significant potential of MWCNTs to enhance CTCM. MWCNTs can span across nanoscale microcracks in the matrix due to its superior bonding with the CTCM. The high stiffness and elastic modulus of the CNTs effectively impede crack propagation and ensure consistent load transfer between microcracks.

In the majority of the inspected regions, MWCNTs were also coated with cement hydrate. As depicted in Fig. 8d, the well-bonded MWCNTs in the cement matrix exhibit a fracture bridging mechanism that helps to prevent crack formation. Additionally, as shown in Fig. 8e,f, MWCNTs form a network structure that highlights their robust connection with the cement matrix.

However, the MWCNTs dispersion effect of GA high concentration G6 sample became worse. The reason is that excessive GA has caused the MWCNTs aggregate again. Resulting in a decrease in CTCM performance. It can be explained from the mechanism of GA dispersed MWCNTs ("Discussion").

#### XRD analysis

**Figure 10:**
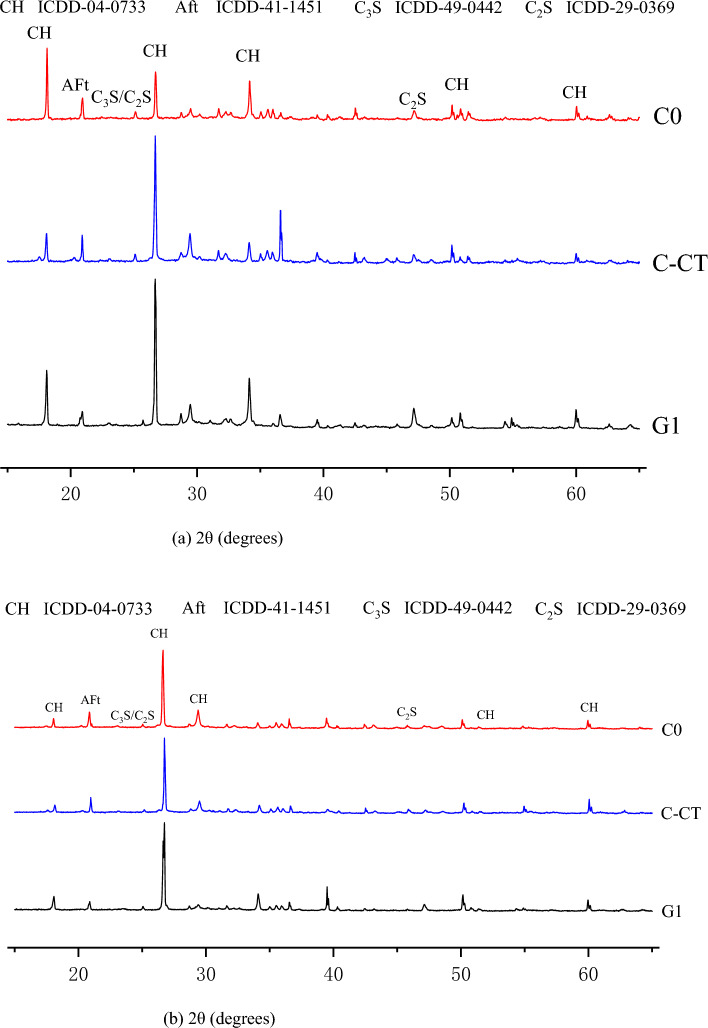
XRD analysis of the C-CT pastes. (**a**) Age: 7 days and (**b**) 28 days.

The crystalline condition of gel products is directly correlated with the mechanical characteristics and durability of cement slurry. The mineral phases of a C-CT slurry having crystalline state and MWCNTs functionalization at various hydration ages were examined using XRD (Rigaku Smart Lab SE, Japan, 10–90°, 2°/min, Cu-Kα ray, 40 kV, 40 mA) patterns. The XRD patterns of C-CT pastes with water-binder ratios of 0.4 are shown in Fig. 10 at ages of 7 and 28 days with MWCNTs (0.08% wt) dispersed with various surfactant concentrations. As can be observed, the G0 sample does not modify the type of hydration products when MWCNTs are added to pure cement.

Calcium hydroxide, dicalcium silicate, hydrated calcium silicate, ettringite, and dolomite are the primary products of hydration. At 28 days, similar hydration products to hydrotalcite were also produced. At 7 and 28 days ages, there is minimal difference in the strength of the primary peak (2θ = 34°) of Ca (OH)_2_ characteristic peak of pure cement when MWCNTs are added, and the strength of the secondary peak (2θ = 18°) increases at first, then declines, and then grows again. The quantity of Ca (OH)_2_ products present in pure cement provides a representation of the hydration products within the system, and it correlates to the strength variation resulting from the incorporation of MWCNTs.

The broad aluminosilicate humps of G1 and C-CT also emerge at distinct points in Fig. 10b (2θ of 18°–27° and 19°–39°), which may be explained by the varied rates of growth of the two three-dimensional amorphous grid structures. When the vitreous in CT was activated by an alkaline activator solution, the hump of G1 broadened and tended to move at a higher angle after the addition of MWCNTs. This suggests that a three-dimensional amorphous network structure was created. However, there was no discernible distinction between G1 and C-CT of the amorphous phase as seen in the XRD patterns of diffraction peaks and large humps. However, it is clear that the phase with the obvious change is crystallin. The performance and efficiency of the resulting C-CT slurry gel can be indicated by any increase in the hump, 2θ range of 18°–27°, which is considered to be significant in the characterization of MWCNTs functionalized C-CT slurry. At the curing age of 7–28 days, the strength and width of the C-S-H peak of G1 are improved. The peak width at 29.4° has improved as a result, and the creation of the C-S-H phase is consistent with the findings of earlier investigations. These internal bonding products developed in the cement matrix including MWCNTs, which were partially enveloped in a needle-like C-S-H type crystal phase. The crystalline octahedral zeolite of zeolite also manifested in all C-CT matrix samples at the curing age of 28 days. With increased reactivity, the amorphous structure transforms into a crystalline structure. The activation of MWCNTs functionalized C-CT composites consumes a significant portion of the Na material, reducing the amount of Na in the pore solution, which leads to the production of sodium aluminosilicate gel.

Due to the decrease of alkalinity, the growth of zeolite is inhibited. In addition, with the increase of curing age, the calcite content in C-CT composite samples increased slightly. This may be because atmospheric CO_2_ is involved in the polymerization reaction. In addition, it can be concluded from Fig. 10 that compared with C-CT, the introduction of MWCNTs increased the C-S-H content of C-CT composite matrix, indicating that the hydration rate increased with the incorporation of MWCNTs.

MWCNTs are employed as C-CT composite matrix nucleation sites, resulting in a large number of reaction sites and the growth of hydration products. This is in line with reports in the literature^49^ that hydration products adhere to the surface of MWCNTs. MWCNTs comprising GA molecule chains are bound by the C-S-H phase via the Ca^2+^ interaction of the pore solution. As a result, the GA concentration on the surface of MWCNTs influences the chain length of the formed C-S-H phase. MWCNTs + GA are regarded as a lead with this reaction throughout the hydration process. It can be speculated that GA activated dispersant solution can improve the compactness of C-S-H gel.

## Discussion

UV–visible spectroscopy and optical microscopy image analysis showed that the optimal blending ratio can be obtained by using the mass ratio of GA: MWCNTs = 1:1 below the critical concentration of MWCNTs dispersion liquid 0.08 wt%. After blending, magnetic stirring was performed for 30 min, and then ultrasonic dispersion was performed for 45 min to produce a uniform single concentration of 0.08 wt% MWCNTs dispersion liquid. The liquid has a very low aggregation area, and is stable and has a long storage time. Through experimental research and analysis, if the GA concentration is adjusted to not meet the GA: CNTs = 1:1 mass ratio, MWCNTs cannot be better dispersed, and the dispersion of MWCNTs becomes worse. In particular, excessive GA concentration is more likely to cause MWCNTs agglomeration.

Hydrophilic polar groups and hydrophobic non-polar groups form surfactant GA. More than 220,000 molecules constitute its molecular weight. It is a combination of some long-chain polymers. It has low viscosity and good adsorption capacity between the two phases. Any two phases can have molecules arranged in the interface layer between them, which supplements the unsaturated force field of the interface to a certain extent and reduces the interfacial tension. Therefore, if the mass ratio is appropriate, GA can increase its hydrophilicity and dispersion by coating MWCNTs with long molecular chains, and a very stable MWCNTs dispersion can be obtained.

**Figure 11:**
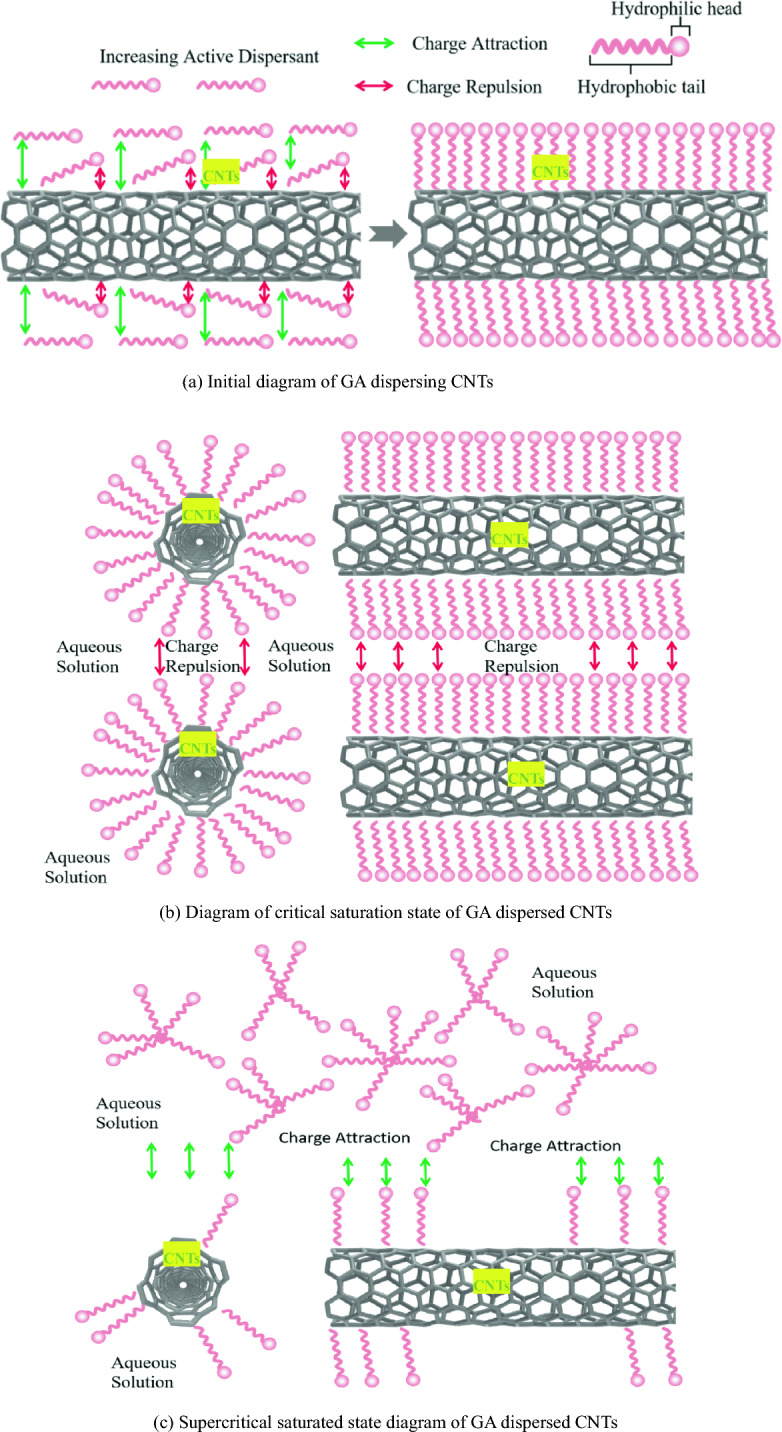
Diagram of GA dispersing CNTs.

The two-stage adsorption model was found to be consistent with the adsorption of GA on the surface of MWCNTs. Due to the adsorption of GA long chains on the surface of MWCNTs, the first short-term adsorption platform was created. The dispersion was low in this concentration range because the lengthy chains of GA molecules laid flat on the surface of MWCNTs, forming a thinner steric hindrance layer (as illustrated in Fig. 11a).

With the gradual increase of GA concentration, a large number of GA molecules are automatically gathered on the surface of MWCNTs. The hydrophilic groups are attracted by highly polar water molecules and try to penetrate into the water, while the hydrophobic groups continue to adsorb on the surface of MWCNTs to form micelles with a certain shape (as shown in Fig. 11b). The contact area between MWCNTs and aqueous solution is reduced, the surface tension of the solution is sharply reduced, and the hydrophilicity of MWCNTs is improved. When the GA concentration reaches about 0.45 g/L, the surface of MWCNTs reaches saturation adsorption, forming a second stable adsorption platform. The spatial steric effect reaches the maximum and the dispersion effect is the best.

The adsorption capacity remained constant when the GA content exceeded 0.45 g/L, however the dispersion performance suffered. This is so that the micelles that are created can be extremely stable in aqueous solution. The amount of micelle and the number of molecules contained in micelle will increase if the concentration of the surfactant GA is increased. Dense micelles have stronger hydrophilicity and lack surface activity, so they cannot further reduce surface tension (as shown in Fig. 11c).

As a consequence of the osmotic pressure between the micelles, MWCNTs will instead begin to aggregate with one another, significantly diminishing the suspension system's particle dispersion capability.

From the dispersion effect of MWCNTs, it can be analyzed that when the concentration of active dispersant GA is low, although GA active molecules can effectively spread on the wall of MWCNTs, there are not enough GA molecules to disperse MWCNTs effectively. However, when a high concentration of GA dispersant is used, the wall of MWCNTs is covered with GA molecules, and the remaining GA active molecules are self-agglomerated. The attraction of the hydrophobic group of the molecular group in the GA molecular self-agglomeration is slightly stronger than that of the hydrophilic group and MWCNTs, and the GA molecules will be 'captured' from the wall of MWCNTs. In this way, the aggregates of GA molecules will absorb the GA molecules that contribute to the dispersion of MWCNTs, and the van der Waals force of MWCNTs will also cause MWCNTs to agglomerate again, and the dispersion effect of MWCNTs will decrease sharply.

In addition, EDS analysis of dispersed MWCNTs showed that there were hydroxyl functional groups on the surface of MWCNTs. Compared with ordinary mortar, adding an appropriate amount of MWCNTs into the copper tailings powder cement slurry can improve the bending and compressive strength. The incorporation of MWCNTs increases the bulk density and reduces the apparent porosity to a certain extent, and the capillary water absorption also proves the positive effect of MWCNTs on the microstructure enhancement. SEM analysis also shows that MWCNTs play a vital role in bridging and passivating cracks, which can improve the physical properties of MWCNTs reinforced copper tailings powder cement paste.

The results showed that the GA hydroxyl functional adsorption layer was formed around MWCNTs due to the attachment of GA hydroxyl functional groups on MWCNTs. This layer forms the microstructure of hydration crystals of modified cement-based materials. In addition, XRD studies found more hydration by-products of MWCNTs modified cement paste. In addition, the results also show that the carbonation resistance of 0.08% MWCNTs modified paste is much higher than that of ordinary cement paste.

## Conclusion

In the present study, the effect of MWCNTs dispersed by GA surfactant on the mechanical properties and microstructure of CTCM composites was investigated. The results of the study show that MWCNTs can promote the hydration of CTCM composites, be beneficial to the evolution of hydration products, crystals and gels in CTCM, and contribute to the densification of the CTCM matrix. Due to the addition of 25% copper tailings powder with insufficient activity in C-CT group, the compressive strength of the 28 days decreased by 17.4%, and the flexural strength of the 28 days decreased by 19.4% compared with the blank group C0. However, due to the enhancement of effectively dispersed carbon nanotubes, the G1 group added with 25% copper tailings powder too, compared with the CT-C group at 28 days, had a 21.5% higher compressive strength and a 20.5% higher flexural strength, both of which were almost reached the mechanical level of the blank group C0. The main conclusions can be drawn as follows:

- The CTCM material replaced 25% cement, and the reduced performance of cement could be compensated by MWCNTs. MWCNTs serve as nanoscale reinforcements in the CTCM matrix to improve the mechanical properties and durability of CTCM, and enhance the strength and stiffness of cement composites.
- At the mass ratio GA:CNTs = 1:1, a high-quality dispersion of MWCNTs aqueous solution with a stable concentration of 0.08% was successfully obtained. The GA hydroxyl functional groups attached to MWCNTs can serve as nucleation sites for calcium silicate hydrate (C-S-H) gel formation.
- When MWCNTs are hydrated in cement, the surface charges of the nanotubes are redistributed. Since the pH value of the CTCM slurry is alkaline, the surface charge of the nanotubes tends to be negative, which promotes the interaction between the surface of the nanotubes and the calcium ions in the slurry to generate more hydroxyl groups. The chemical bonding between them plays an important role. This is also the main factor for the enhancement of CTCM strength.
- Dispersion and stable MWCNTs in CTCM can be used as seeds for the formation of hydration products to exert nanoscale strength, which can effectively improve the mechanical properties and durability of concrete.
- GA dispersants are environmentally friendly. Compared with other surfactants and dispersants, GA is a natural plant acacia secretion, derived from plants, has good biodegradability, and is environmentally friendly. And GA is relatively cheap and readily available, making it a cost-effective option for improving the properties of cement-based materials.

Moreover, in CTCM materials, the activity of CT is insufficient. We recommend that future researchers improve the hydration of the slurry, try to investigate the excitation effect of alkali activators on CTCM, and study the synergistic enhancement of alkali activators and CNTs performance.

### Supplementary Information

Supplementary Information.

## Data Availability

The datasets used and/or analyzed during the current study available from the corresponding author on reasonable request.

## Abbreviations

CTCM: Copper tailing-based cementitious material
MWCNTs: Multi-walled carbon nanotubes
CNTs: Carbon nanotubes
GP: Geopolymer
CTGP: Copper tailings geopolymer
GPC: Geopolymer concrete
GA: Arabic gum
PVP: Polyvinylpyrrolidone
SDBS: Sodium dodecyl benzene sulfonate
OPC: Ordinary Portland cement
CT: Copper tailings powder
CTP: Copper tailings powder
C-CT: Copper tailing powder cement paste
C0: Pure cement paste
UHPC: Ultra-high-performance concrete
G1: The specimens of mass ratio GA: MWCNTs = 1:1 of C-CT
G2: The specimens of mass ratio GA: MWCNTs = 2:1 of C-CT
G3: The specimens of mass ratio GA: MWCNTs = 3:1 of C-CT
G4: The specimens of mass ratio GA: MWCNTs = 4:1 of C-CT
G5: The specimens of mass ratio GA: MWCNTs = 5:1 of C-CT
G6: The specimens of mass ratio GA: MWCNTs = 6:1 of C-CT
G0: The specimens without active dispersant of C-CT
C-S-H: Hydrated calcium silicate gel
GB/T: Chinese standard
JCJ/T: Chinese standard
W/B: Water-binder ratio
RH: Relative humidity
