# Supplementary material for: MWCNTs dispersion adopting GA and its application towards copper tailings-based cementitious materials
Source: Sci Rep. 2023 Sep 26;13:16081. doi: 10.1038/s41598-023-43133-7 (PMC10522576; doi:10.1038/s41598-023-43133-7)
Supplement: Supplementary file 1 — Supplementary Information. [file 41598_2023_43133_MOESM1_ESM.docx]

Main technical parameters of SEM and EDS

Main technical parameters of SEM.

Electron gun: Schottky field emission electron gun; resolution: 1.0nm @ 15kV, 1.6nm @ 1kV; electron optical path: electron beam in the barrel without cross optical path acceleration voltage: 0.02-30kV, 10V step continuously adjustable probe beam: 3pA-20nA, stability is better than 0.2 % / h magnification : 10x-1,000. 000x objective lens: electromagnetic / electrostatic composite lens detector: INLENS and ET secondary electron detector.

Main technical parameters of EDS.

**Response:** Main technical parameters of EDS: EDS spectrometer Energy spectrum model : Smartedx energy spectrum analysis working distance : 8.5mm sample chamber size : 365mm x 275mm sample stage stroke : X = 125mm ; y = 125mm ; z = 50mm ; t = -10 ° to 90 ° ; r = 360 ° ( continuously adjustable ) Image acquisition : up to 32k x 24k Test reference steps : take a small amount of sample / block / film sample directly to the conductive adhesive, and use the Quorum SC7620 sputtering coating instrument to spray gold for 45s ( the specific spraying time is determined according to the sample / test requirements ), spraying gold is 10mA ; subsequently, the ZEISS Sigma 300 scanning electron microscope was used to photograph the sample morphology and energy spectrum mapping. The acceleration voltage was 3kV when the morphology was photographed, and the acceleration voltage was 15kV when the energy spectrum mapping was photographed. The detector was SE2 secondary electron detector.
